# Fruit Quality Classification Through Feature Fusion and Deep Learning

**DOI:** 10.3390/foods15203624

**Published:** 2026-10-12

**Authors:** Shajia Nafees, Muhammad Javed Iqbal, Iftikhar Ahmad, Madini O. Alassafi, Neelum Noreen

**Affiliations:** 1 Department of Computer Science, University of Engineering and Technology, Taxila 47050, Pakistan; shajianafees2@gmail.com (S.N.); javed.iqbal@uettaxila.edu.pk (M.J.I.); 2 Department of Information Technology, Faculty of Computing and Information Technology, King Abdulaziz University, Jeddah 21589, Saudi Arabia; malasafia@kau.edu.sa; 3 School of Information Technology, Whitecliffe College, Auckland 1010, New Zealand; neelumn@whitecliffe.ac.nz

**Keywords:** fruit quality classification, ResNet50V2, CNN, deep learning, data augmentation, HOG, LBP

## Abstract

Fruit quality assessment is an integral part of food safety, loss reduction after harvest and consumer satisfaction. In modern agricultural production systems, where quality control is essential for ensuring both domestic and export markets, it continues to play a vital role. Manual grading, which is widely used, is poorly time-efficient, imprecise, and prone to human error, and is reliant upon the human eye and human skill. Such limiting practices can result in variability and, in large-scale production, high operating expenses. In this paper, we propose a hybrid classification model using deep learning features extracted from ResNet50V2 along with traditional handcrafted features such as Histogram of Gradients (HOG), Local Binary Patterns (LBP), and color, texture and shape descriptors. The purpose of this study is to develop a model to combine high-level and low-level feature representation to gain a comprehensive understanding of fruit quality characteristics. The proposed preprocessing technique will be followed by data augmentation, ANOVA-based feature selection, and classification using K-Nearest Neighbors (KNN), Random Forest (RF) and Extreme Gradient Boosting (XGBoost). This multi-stage course of action is specifically designed so that the extracted features are appropriate, relevant and discriminative for correct classification. For experimentation, standard datasets were chosen as FruitNet and tomato. There are six fruit types included in the FruitNet dataset with three different quality classes, and another tomato dataset with three ripeness-based classes. XGBoost always outperforms other classifiers on the FruitNet dataset with an accuracy of 97.3% and on the tomato dataset with an accuracy of 92%. The findings validate the validity of the proposed model and its use in all crops under all grading systems.

## 1. Introduction

To meet rising consumer expectations, the global food supply chain is progressively focused on delivering safe and high-quality fruits. However, usual fruit quality grading based on manual examination suffers from subjectivity, fatigue, and inconsistency [1]. Nearly 45% of global fruit and vegetables are wasted, representing a significant economic and resource burden by these inefficiencies [2].

Developments in computer vision and artificial intelligence have paved the way for the advancement of automated grading systems. Convolutional Neural Networks (CNNs) have been used to extract the classified image features, whereas handcrafted features capture fine-grained visual patterns [3,4]. Regardless of this progress, issues such as dataset imbalance, visual variability, and inter-class similarity remain challenges to widespread deployment [5].

Likewise, the increase in demand for export-quality fruit production, food safety regulatory submission, and consumer inclination for visually attractive fruits further highlights the need for robust automated systems [6,7,8]. Machine learning and deep learning frameworks have showed their ability to solve the aforementioned problems [9,10]; however, scalability among diverse fruit types and real-world environmental conditions remains a key research challenge [11,12,13]. By utilizing different learning algorithms, these techniques can classify complex relationships as well as underlying structures inside data that may be complex for detecting the use of conventional statistical methods [14].

Recent developments in machine learning as well as deep learning have allowed scientists to develop sophisticated models capable of identifying nonlinear relationships and sophisticated shapes in large-dimensional datasets [15,16,17]. Neural networks, specifically, can automatically learn hierarchical representations from sophisticated and high-dimensional data, as well as classification, enabling prediction of effectiveness through a broad range of domains of application [18,19].

A real-world solution must be accurate, computationally efficient, and adaptable to varying production environments.

In this paper, we have addressed the challenges by proposing a hybrid framework that fuses deep learning with handcrafted features and integrates robust preprocessing and augmentation, feature fusion and machine learning-based classification. The main contributions of this work are as follows:

- Development of a hybrid feature extraction and fusion pipeline that combines ResNet50V2 deep features with HOG, LBP, and color- and shape-based handcrafted features.
- Evaluation of the proposed pipeline on two fruit quality datasets with different class structures and levels of complexity to examine its classification performance under different data characteristics.
- Analysis of the impact of dataset size, class imbalance, and feature fusion on the classification accuracy.

Fruit quality assessment has been an active research area for decades, with researchers proposing diverse techniques ranging from handcrafted image-processing methods to modern deep learning frameworks. Early contributions focused on extracting basic color, shape, and texture features, whereas more recent studies have explored Convolutional Neural Networks (CNNs), transfer learning, and hybrid approaches to improve robustness and generalization.

Bhargava and Bansal [20,21] developed a machine learning system for the automatic detection and grading of multiple fruits, where Support Vector Machine (SVM) outperformed other classifiers with 95.72% accuracy. After that, they focused on apple quality evaluation using Support Vector Machine classifiers and obtained accuracies of 96.81% and 93.00% on two datasets. Similarly, Long and Thinh [22] proposed a machine learning-based grading of mangoes using external features, where Random Forest achieved 98.1% accuracy.

Extending these advances, Nasiri et al. [23] proposed a CNN-based model using VGG-16 architecture for automated classification of date fruits into different ripening stages and defective categories, achieving an accuracy of 96.98%. Similarly, Fan et al. [24] combined a CNN with a computer vision system for the real-time detection of defective apples, achieving 96.5% accuracy. Deep learning methods have gained momentum [25], as Foong et al. [26] applied ResNet50 for rotten fruit detection of apple, orange, and banana with 98.89% accuracy. In parallel, Miah et al. [27] compared five CNN models and reported that InceptionV3 achieved 97.34% accuracy in fresh/rotten fruit detection. Al Haque et al. [28] developed CNN models for banana strain detection and freshness evaluation, achieving 93.4% classification accuracy and 98.3% in rotten banana detection. Similarly, Sharma and Kumar [29] applied ResNet50 for fresh/rotten fruit classification on six fruit classes with an overall accuracy of 95%.

More recently, Zha et al. [30] used transfer learning with ResNet50 and SVM for red grape appearance grading, achieving an accuracy of 95.08%. Similarly, Knott et al. [31] employed Vision Transformers for fruit quality assessment, matching CNN performance with fewer training samples, and improving accessibility.

Hybrid and ensemble frameworks have also shown promise in this regard [9]. Mputu et al. [32] proposed a CNN-SVM model for tomato quality grading, achieving 97.50% binary classification accuracy and 96.67% multiclass accuracy. Mallegowda et al. [33] applied CNN with VGG16 transfer learning for fruit freshness classification, achieving over 90% accuracy. Similarly, Tai et al. [34] presented an ensemble learning framework for mango grading, with stacking models achieving 98.63% accuracy. Akram et al. [35] developed a machine vision-based system combining CNN and image processing for mango and tomato grading, with validation accuracies of 95% and 94%, respectively.

Overall, these studies collectively demonstrate a shift from handcrafted features to deep learning and hybrid techniques, with consistently improving accuracies as shown in Table 1. However, many studies remain limited to specific fruits or controlled environments, highlighting the need for generalizable frameworks that can handle diverse datasets and variability, a gap that the present research aims to address.

## 2. Materials and Methods

### 2.1. Dataset Description

The proposed methodology was evaluated using two datasets. These datasets provide complementary image samples with different fruit types, quality categories, acquisition conditions, and visual variations.

**Figure 1:**
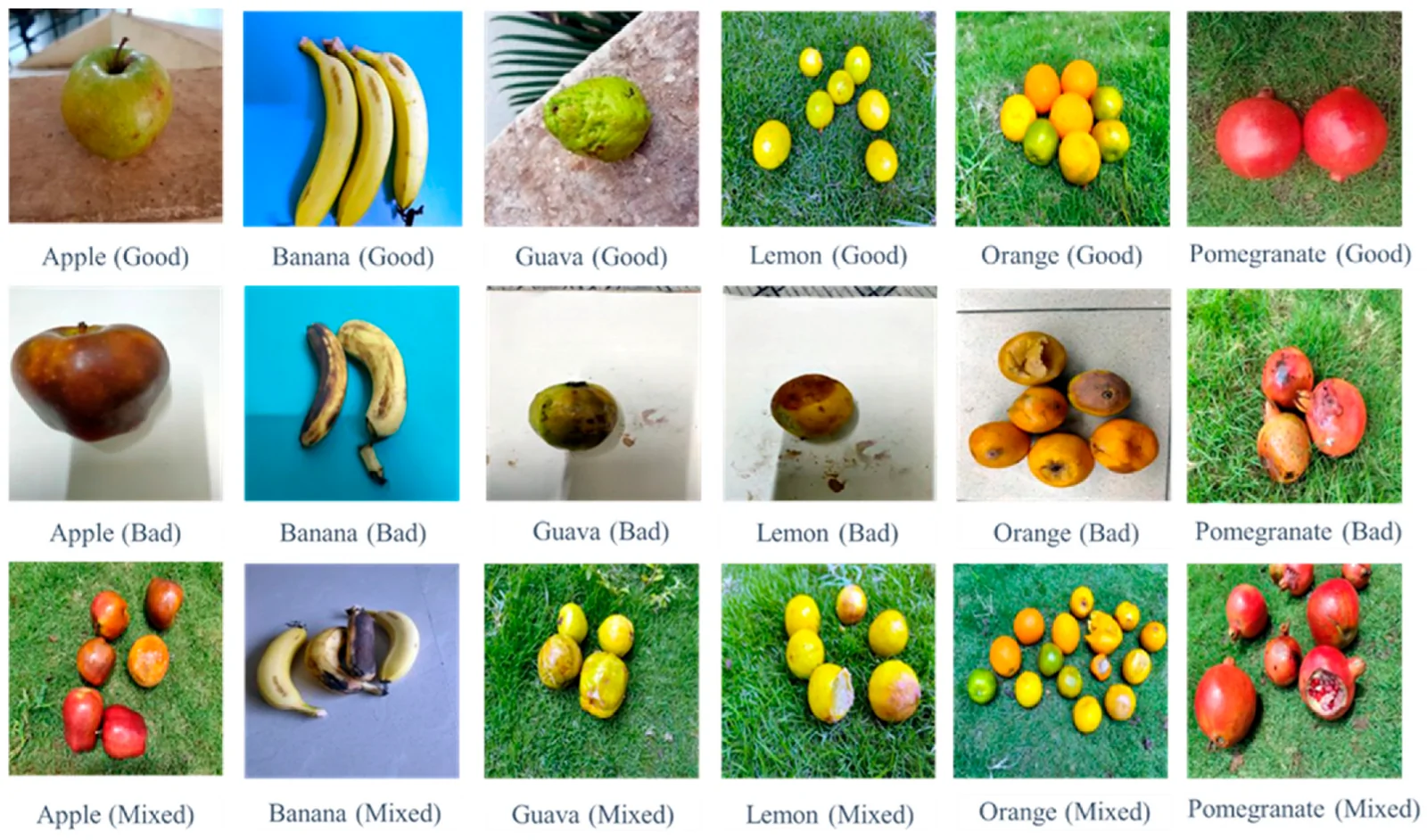
Illustration of FruitNet dataset.

**Figure 2:**
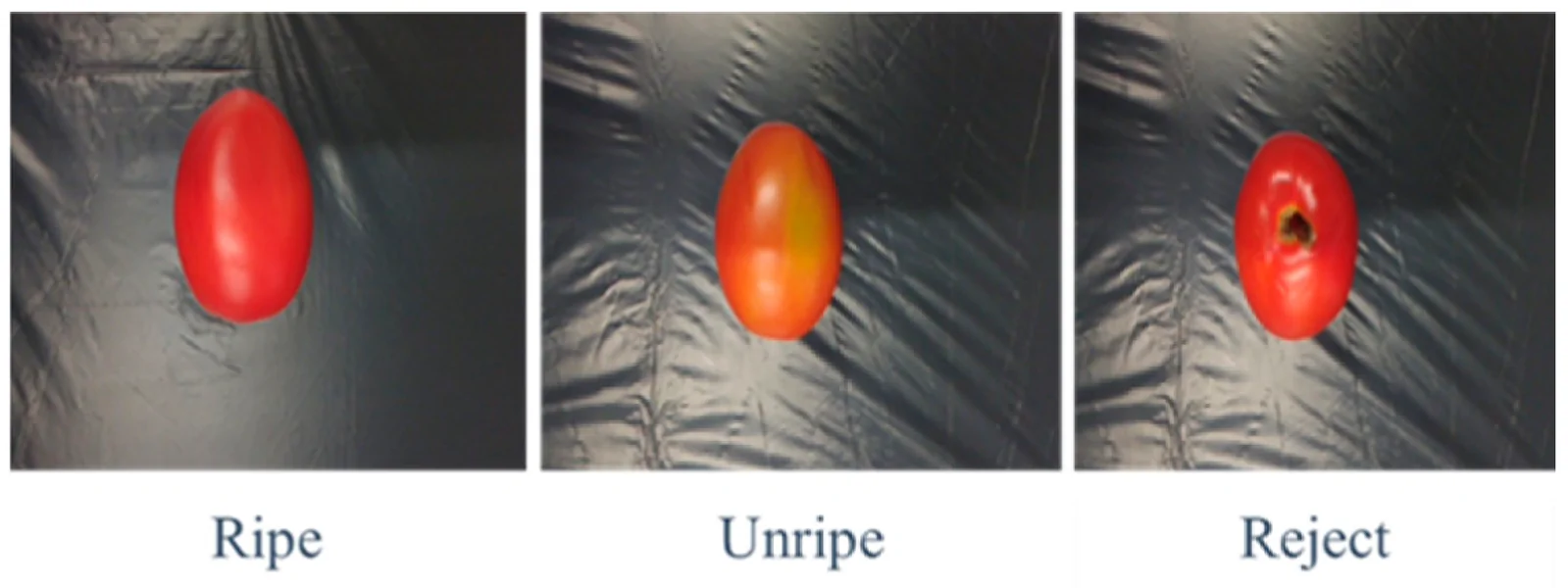
Illustration of tomato dataset.

- FruitNet Dataset [36]: It is a publicly available dataset built for the purpose of fruit classification and quality assessment. It comprises 19,500+ images of six fruits (apple, banana, guava, lime, orange, and pomegranate) with three classes (Good, Bad, and Mixed) as shown in Figure 1. The Good-quality fruit class has 11,664 images, the Bad-quality fruit class has 6778 images, and the Mixed-quality fruit class has 1074 images. This dataset was originally created using a high-resolution mobile phone camera. The images were captured under different backgrounds (dark-colored, light-colored, grass, ground, etc.), angles and lighting conditions, providing variations in illumination, visual appearance, fruit orientation and viewpoint including front, top, backward, bottom and rotated views.
- Tomato Dataset [32]: It is also a publicly available dataset which was used to further evaluate the proposed framework for tomato quality classification. It contains 2400 images with 3 classes (Ripe, Unripe, and Reject) as shown in Figure 2. Each class of tomato consists of 800 images. These images were obtained from 600 tomatoes, with four images captured from each tomato. The four views correspond to the top, bottom, rear, and front sides of the tomato. The tomatoes exhibit variations in size, shape, color, and visible defects. The images were acquired using an NVIDIA Jetson TX1 single-board computer equipped with an onboard RGB camera. Image acquisition was performed under an uncontrolled lighting environment, with a fixed distance of approximately 20 cm between the camera and the table surface. The tomato images were categorized according to the quality criteria described in the original dataset study, which followed OECD and USDA standards.

In the present study, the original quality labels provided with both the datasets were used, and no new quality labels were manually assigned.

### 2.2. Proposed Methodology

**Figure 3:**
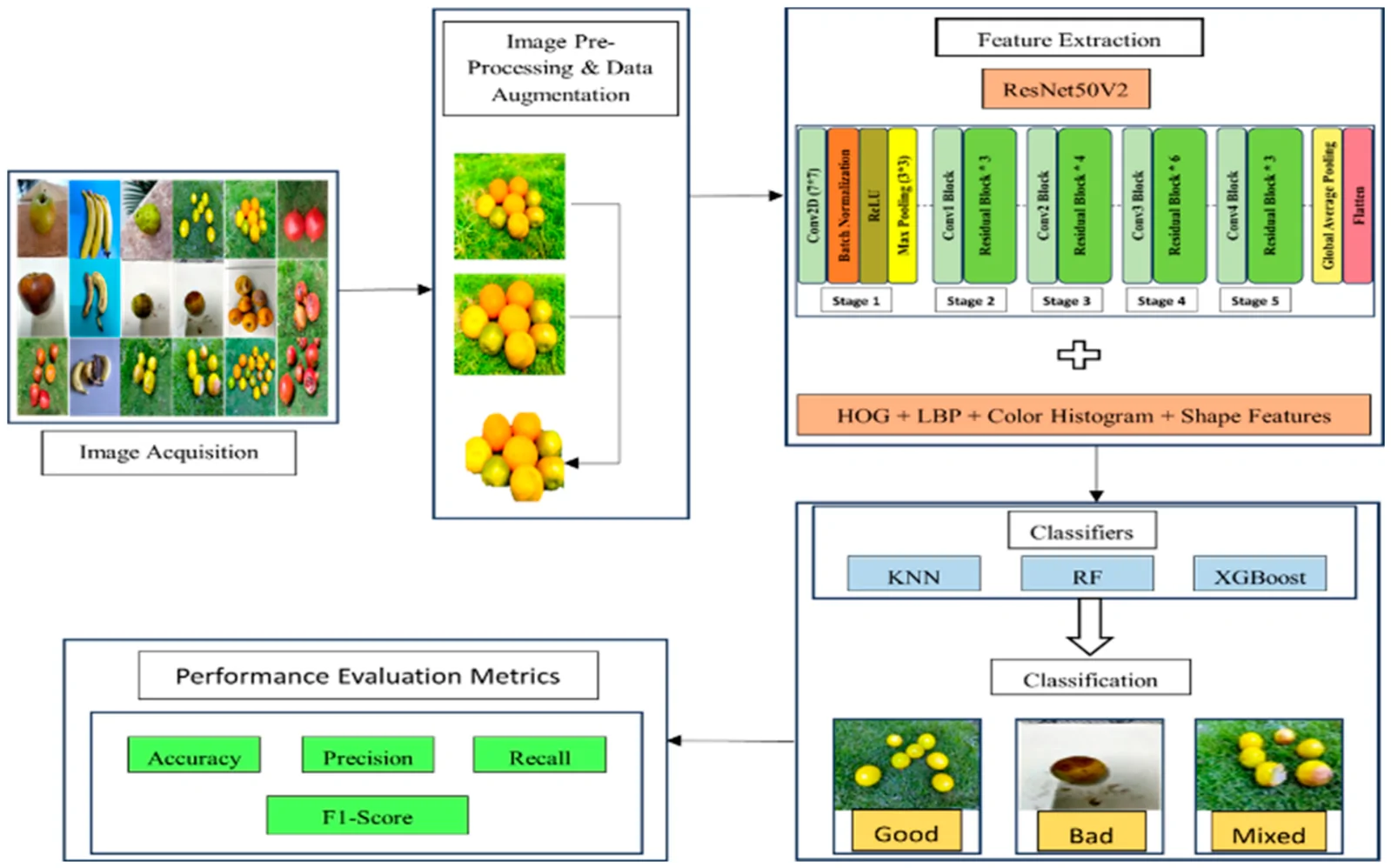
Proposed methodology.

The proposed methodology involves a multi-stage pipeline that begins with preprocessing including image resizing, color space conversion and normalization. To ensure reliable model validation and to minimize the risk of data leakage, the dataset was divided into training, validation, and test subsets using stratified splitting, with 64% of the images used for training, 16% for validation, and 20% for testing. Then data augmentation was applied only to the training subset to increase the variability of the training data, whereas the validation and test subsets were kept unaugmented. Feature extraction was then performed using the ResNet50V2 deep learning model along with the computer vision-based HOG, LBP, color and shape descriptors, after which the extracted features were fused to form a hybrid feature representation. Following feature fusion, feature standardization was performed by fitting the StandardScaler exclusively on the training features, which was then used to transform the validation and test features. Similarly, the ANOVA-based SelectKBest feature selection was applied by fitting only on the training data and subsequently applied to the validation and test sets. This feature selection was used to optimize the dimensionality and retain the most discriminative features, and then the multiple classifiers were used for prediction. The workflow of this system is illustrated in Figure 3.

#### 2.2.1. Image Preprocessing

In the preprocessing stage, fruit images were resized to fixed dimensions of 128 × 128 for consistency. Color space conversion was then applied, which converted the RGB to HSV and LAB color spaces to highlight informative visual details, while normalization scaled pixel intensities within a uniform range of [0, 1]. These steps lessen the variations due to scale, radiance, and contrast, creating a normalized input for subsequent processing.

#### 2.2.2. Data Augmentation

Data augmentation [37] was used to boost diversity and decrease the overfitting. It was applied in this proposed methodology after splitting the dataset into training, validation and testing subsets. Augmentation was performed only on the training dataset, while the validation and testing datasets were kept unaugmented to prevent information leakage and ensure an unbiased evaluation of the proposed model. On-the-fly augmentation using Keras’ ImageDataGenerator [38] established random transformations such as rotation, flipping, scaling, and brightness adjustment, which are shown in Table 2. This method prevented storing extra copies, enhanced the GPU memory, and ensured that the model learned robust features from non-stop variations.

#### 2.2.3. Feature Extraction and Fusion

In this paper, a hybrid feature extraction approach was employed that fused deep learning and handcrafted descriptors.

1. ResNet50V2 (Deep Features):

ResNet50V2 [39], a 50-layer residual CNN pretrained on ImageNet, was used for extracting deep features by eliminating the top layer and obtaining 2048-dimensional vectors from the Global Average Pooling output.

(1) f_DL_ = ϕ_ResNet50V2_(X)ϵR^2048^

In Equation (1), ϕ_ResNet50V2_ denotes the feature extraction function applied to the input image X. These features capture robust patterns, such as edges, textures, and object shapes.

**Figure 4:**
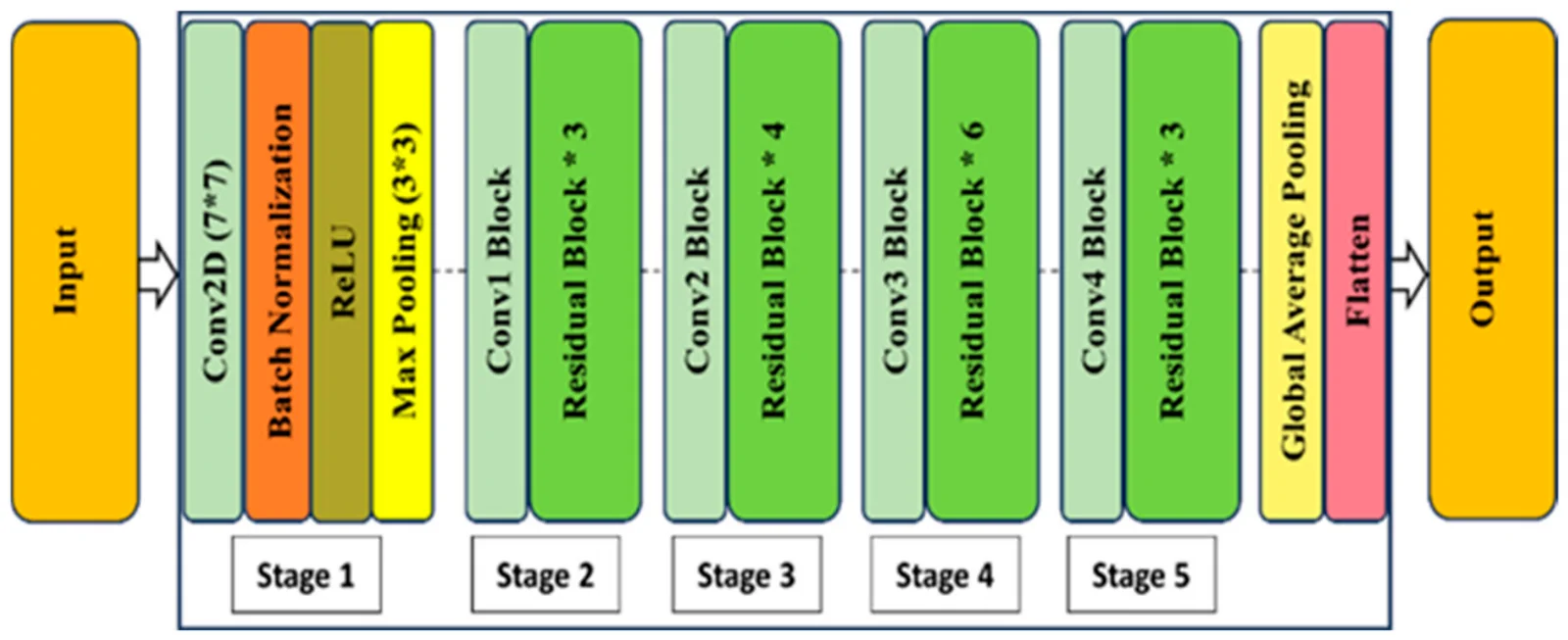
Architecture of ResNet50V2.

The architecture of ResNet50V2 is illustrated in Figure 4. Each residual block in the architecture consists of three convolutional layers with kernel sizes of 1 × 1, 3 × 3, and 1 × 1.

2. Histogram of Oriented Gradients (HOG):

HOG features capture the gradient orientation and magnitude of edges, which are effective for identifying contours and surface defects [40]. The gradient of an image I(x,y) is computed as

(2) G_x_ = I(x + 1,y) − I(x − 1,y)

(3) G_y_ = I(x,y + 1) − I(x,y − 1)

By Equations (2) and (3), the orientation θ and magnitude M are

(4) $$\theta (x,y)=\tan^{-1}\left(\frac{G_{y}}{G_{x}}\right)$$

(5) $$M(x,y)=\sqrt{{G_{x}}^{2}+{G_{y}}^{2}}$$

The feature vector for HOG is shown in Equation (6) by using Equations (4) and (5).

(6) $$f_{\mathrm{HOG}}\left(I\right)=\underset{c\in \mathrm{cells}}{⋃}\mathrm{Hist}\left(\theta \left(x,y\right),M\left(x,y\right)\right)\forall (x,y)\in c$$

These descriptors aided in the detection of irregular textures and blemishes on fruit surfaces.

3. Local Binary Patterns (LBP):

LBP encodes pixel intensity relationships to represent fine-grained textures [41]. For a pixel p_c_ with neighbors p_i_, the LBP pattern is shown in Equation (7).

(7) $$\mathrm{LBP}\left(p_{c}\right)=\sum_{i=0}^{P-1}s\left(p_{i}-p_{c}\right)\cdot 2^{i}$$

where $s(x)=\left\{\begin{array}{c} 1\;\mathrm{if}\;x\geq 0\\ 0\;\mathrm{otherwise} \end{array}\right.$ and the feature vector for LBP is shown in Equation (8).

(8) $$f_{\mathrm{LBP}}(I)=\mathrm{Hist}({\left\{\mathrm{LBP}(p_{c})\right\}}_{p_{c}\in I})$$

This was useful in distinguishing smooth fruit skin from bruised or decayed regions, thereby aiding in decay detection.

4. Color Histograms:

Color histograms from the RGB, HSV, and LAB spaces provide statistical information regarding intensity distributions [42]. As ripeness and decay are strongly color-dependent, these features effectively capture maturity levels and discoloration.

For a given color space C with channels, c ∈ {c_1_,c_2_,…,c_k_}:

(9) $$f_{\mathrm{Color}}(I)=\underset{c\in C}{⋃}\mathrm{Hist}(I_{c})$$

In Equation (9), Hist(I_c_) computes the normalized histogram of pixel intensities in the channel c.

5. Shape Descriptors:

Shape-based features, including area, perimeter, aspect ratio, and solidity, were extracted from the contours [43], as shown in Equation (10).

(10) f_Shape_ (I) = {A,P,Aspect Ratio,Extent,Solidity}

where A = Area of object, P = Perimeter of object, $\mathrm{Aspect Ratio}=\frac{\mathrm{width}}{\mathrm{height}}$, $\mathrm{Extent}=\frac{\mathrm{Area of object}}{\mathrm{Bounding box area}}$, and $\mathrm{Solidity}=\frac{\mathrm{Area of object}}{\mathrm{Convex hull area}}$.

These descriptors distinguish healthy fruits from irregular ones affected by damage or decay.

6. Hybrid Feature Fusion:

All obtained features (deep and handcrafted) were fused into a single hybrid feature vector, as shown in Equation (11).

(11) f_hybrid_ = [f_DL_∣∣f_HOG_∣∣f_LBP_∣∣f_Color_∣∣f_Shape_]

This multi-scale interpretation deepened the dataset, refining robustness and ensuring better classification accuracy throughout various fruit conditions.

#### 2.2.4. Feature Scaling and Selection

Because the hybrid feature vector held diverse scales, regularization was applied to ensure a consistent contribution during learning. Each feature dimension was normalized as shown in Equation (12).

(12) $$\tilde{f}=\frac{f_{\mathrm{hybrid}}-\mu }{\sigma }$$

where μ and σ are the mean and standard deviation of the feature column, respectively.

To reduce dimensionality, the discriminative strength of each feature was evaluated using the ANOVA F-test [44], where the F-score for the feature j is shown in Equation (13).

(13) $$F_{j}=\frac{\mathrm{variance between classes of feature j}}{\mathrm{variance within classes of feature j}}$$

Features with higher *F_j_* values were ranked as more relevant, and the SelectKBest method retained the top 100 features. The value of K = 100 was selected based on the observed trade-off between classification performance and computational cost during model development. A smaller feature set (K = 50) resulted in a slight reduction in accuracy, whereas increasing K to 200 did not provide a noticeable accuracy improvement but increased the computational/training cost; therefore, K = 100 was retained as a balanced choice. In Equation (14), the final vector z is shown.

(14) $$z={\mathrm{SelectKBest}}_{100}(\tilde{f},\{F_{j}\})$$

This selection step reduces the training complexity while preserving the most discriminative information for classification.

#### 2.2.5. Classification

The final hybrid feature vector z was used to train supervised classifiers for fruit quality assessment. Three distinct machine learning models, namely KNN, Random Forest (RF), and XGBoost, were implemented and compared. The dataset was split into training, validation and testing sets in a stratified manner to preserve the class balance. Encoded labels and class weights addressed categorical outputs and imbalance, ensuring fair learning across all classes. The classification task can be expressed in a generalized form, as shown in Equation (15).

(15) $$\hat{y}=C(z)$$

where C denotes the classification function (KNN, RF, or XGBoost) mapping the feature space to the predicted class label $\hat{y}$.

1. K-Nearest Neighbors (KNN):

KNN [45] was employed as the baseline classifier for the selected hybrid feature vector z. The specific mapping is expressed as shown in Equation (16).

(16) $$\hat{y}=\mathrm{KNN}(z)$$

With K = 5, it performed relatively well, particularly on the tomato dataset, where class boundaries were evident, but struggled with uncertain categories in the FruitNet dataset.

2. Random Forest (RF):

Random Forest [46] controls an ensemble of 100 decision trees with Gini impurity as the split measure. Its estimation can be denoted as shown in Equation (17).

(17) $$\hat{y}=\mathrm{RF}(z)$$

Its ensemble quality enabled strong performance, specifically in well-separated classes such as “Good Quality” and “Ripe,” while preserving robustness against noisy and high-dimensional features.

3. Extreme Gradient Boosting (XGBoost):

XGBoost [47] outperformed the other classifiers, remarkably in challenging classes such as “Mixed Quality” and “Reject.” Set up with n_estimators = 200, learning rate = 0.1, and early stopping, its estimation is expressed as shown in Equation (18).

(18) $$\hat{y}=\mathrm{XGB}(z)$$

This formulation efficiently managed complex feature interactions and determined strong generalization across both the datasets.

## 3. Results

The proposed hybrid approach was assessed on two benchmark datasets, FruitNet and tomato, using three classifiers: K-Nearest Neighbors (KNN), Random Forest (RF), and Extreme Gradient Boosting (XGBoost). Performance was evaluated via standard evaluation metrics, including accuracy, precision, recall, F1-score, and confusion matrices [48,49].

### 3.1. Results on FruitNet Dataset

The FruitNet dataset consists of multi-class fruit quality images classified into *Good*, *Bad*, and *Mixed* classes. Table 3 summarizes the classification performance across the models.

**Figure 5:**
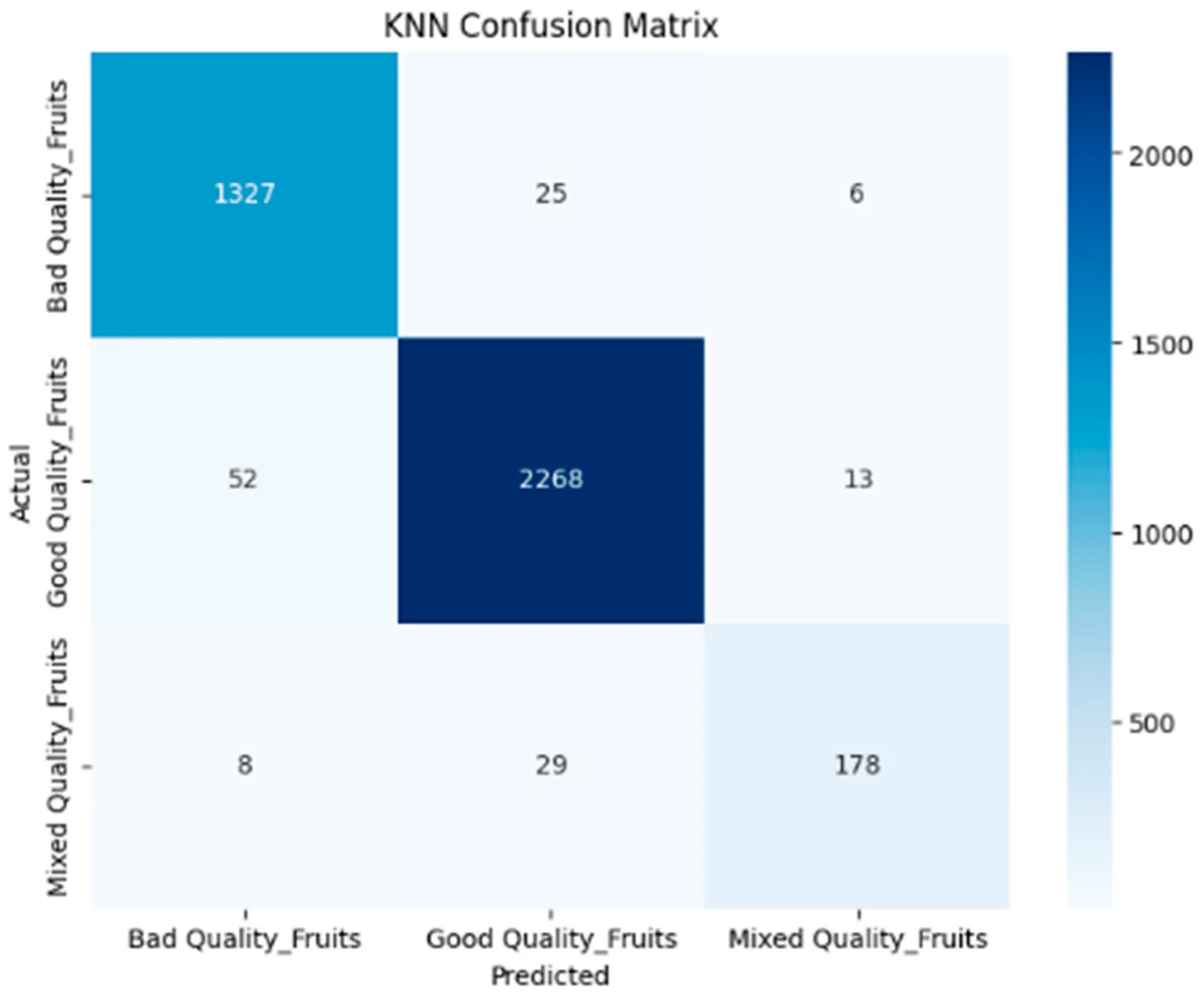
KNN confusion matrix for FruitNet dataset.

**Figure 6:**
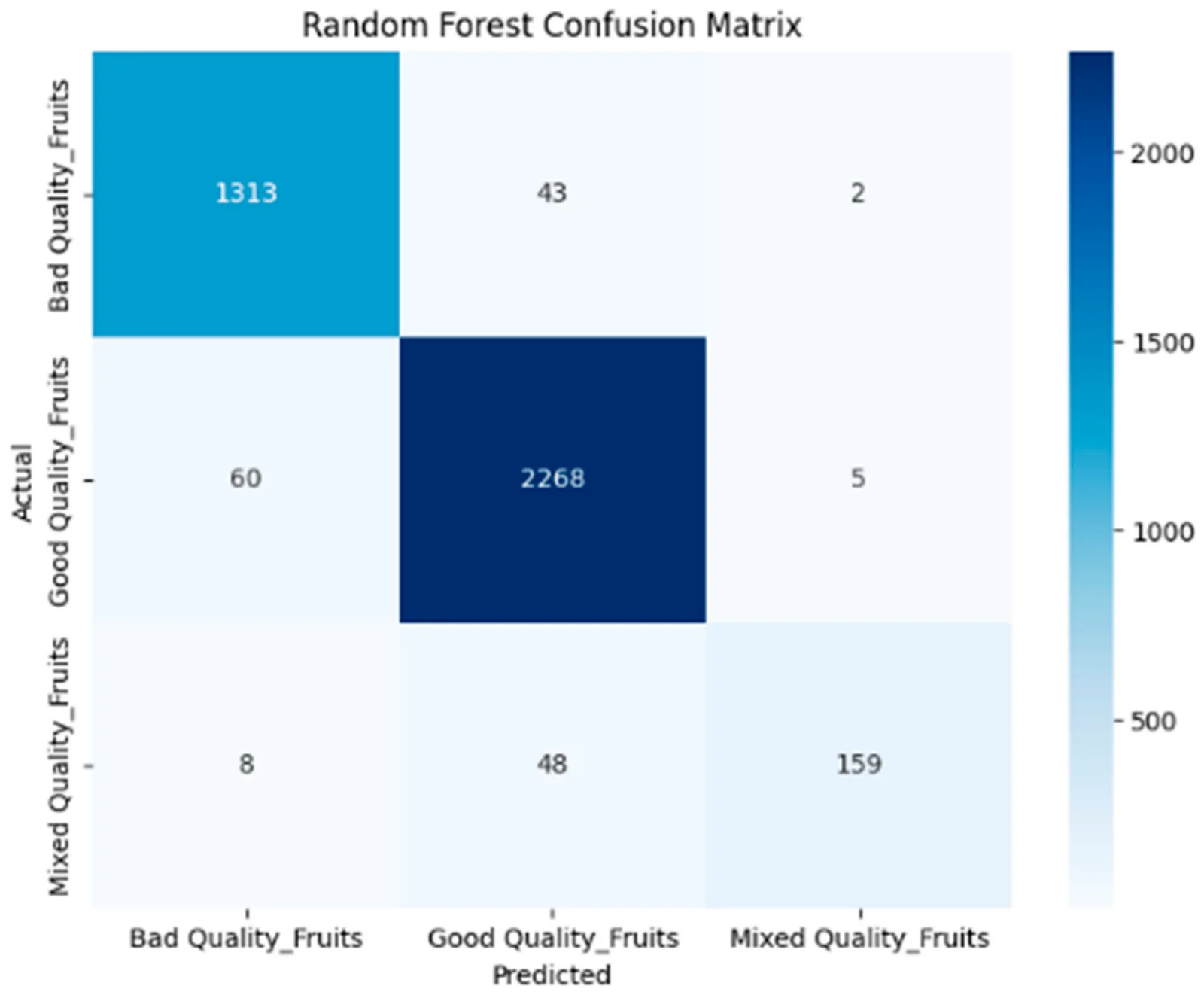
RF confusion matrix for FruitNet dataset.

**Figure 7:**
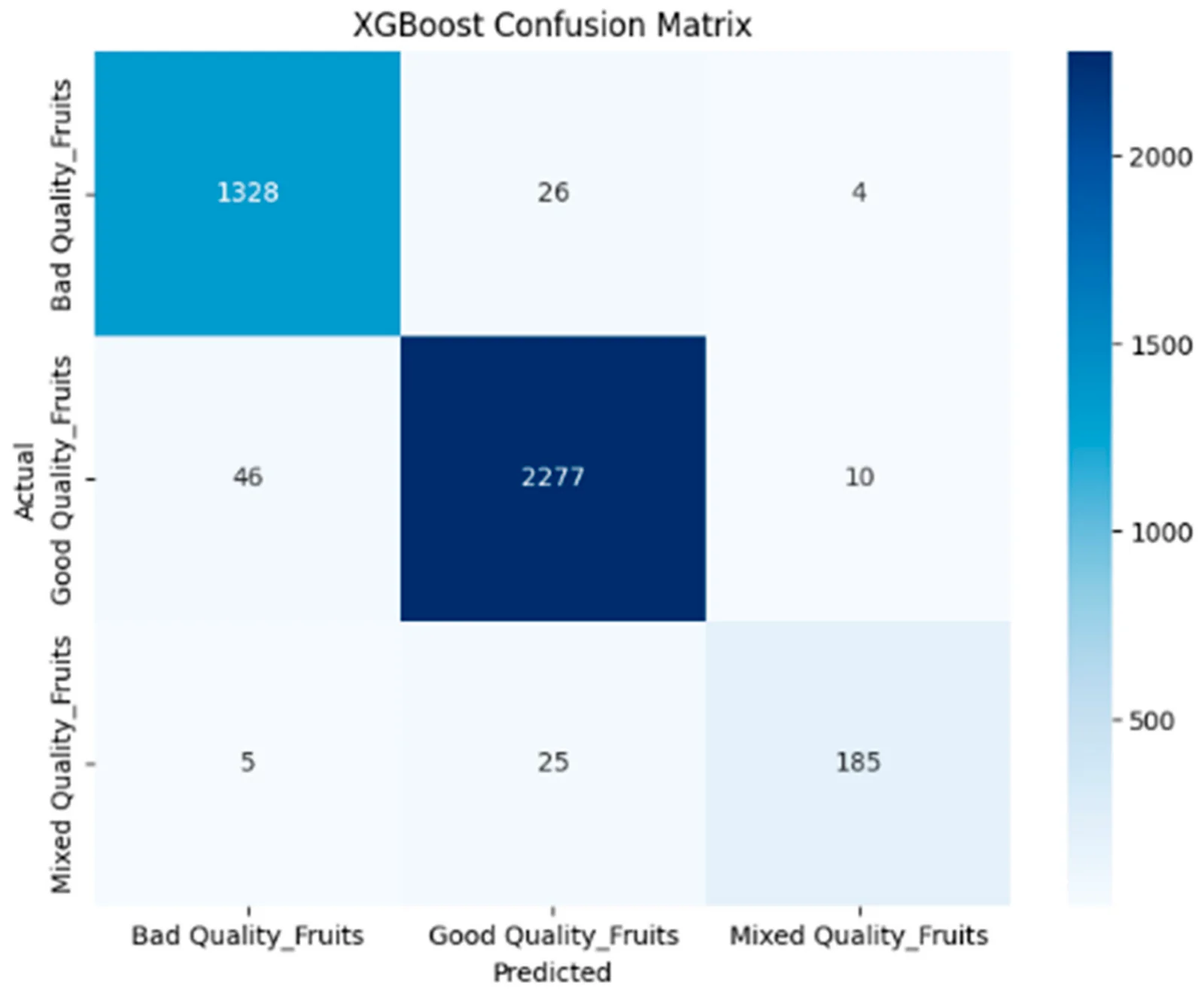
XGBoost confusion matrix for FruitNet dataset.

As observed, XGBoost consistently outperformed RF and KNN, achieving the highest accuracy and F1-score. The confusion matrices shown in Figure 5, Figure 6 and Figure 7 further highlight that the “Mixed Quality” class posed the most challenges, with misclassifications occurring mainly between *Good* and *Mixed*.

**Figure 8:**
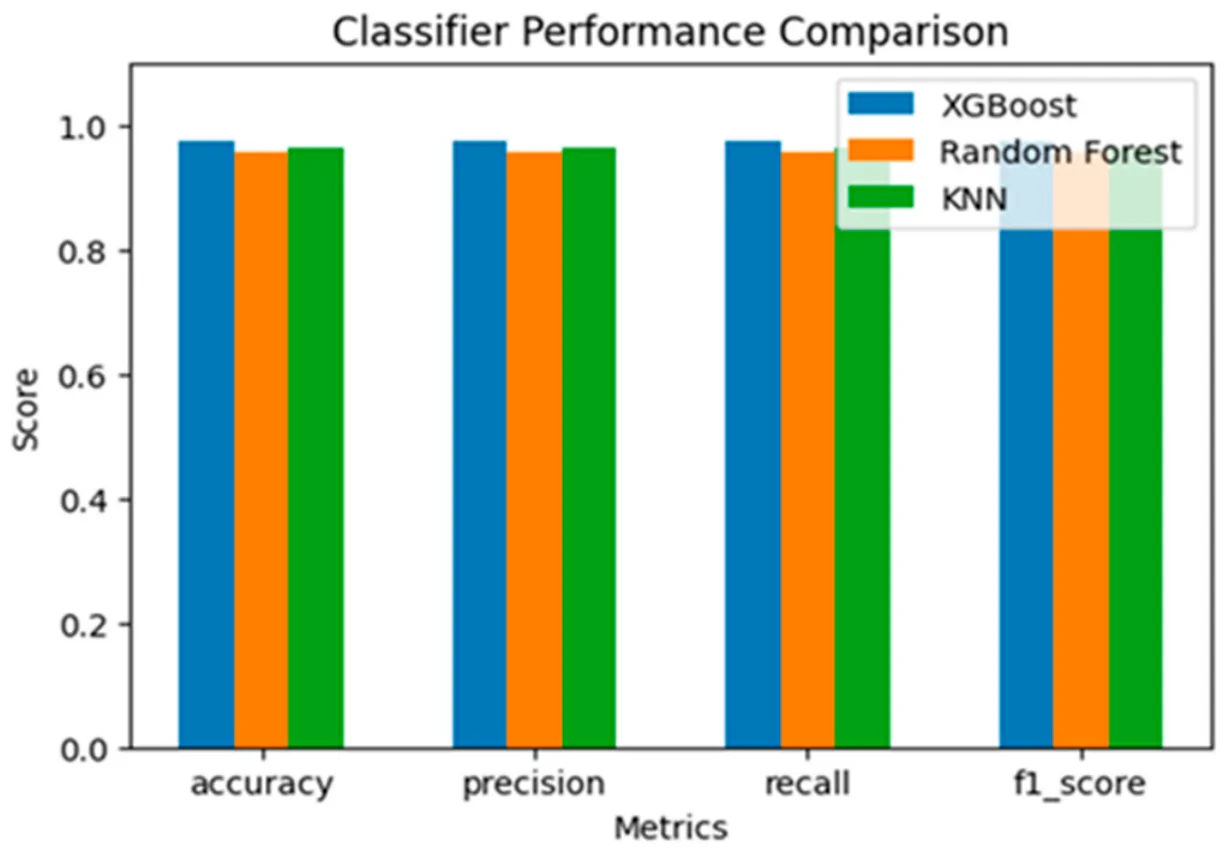
Classifiers’ performance comparison for FruitNet dataset.

Figure 8 shows a comparison of all classifiers used in this research to evaluate the FruitNet dataset.

### 3.2. Results on the Tomato Dataset

The tomato dataset was divided into *Ripe*, *Unripe*, and *Reject* categories. Table 4 presents the model’s performance.

**Figure 9:**
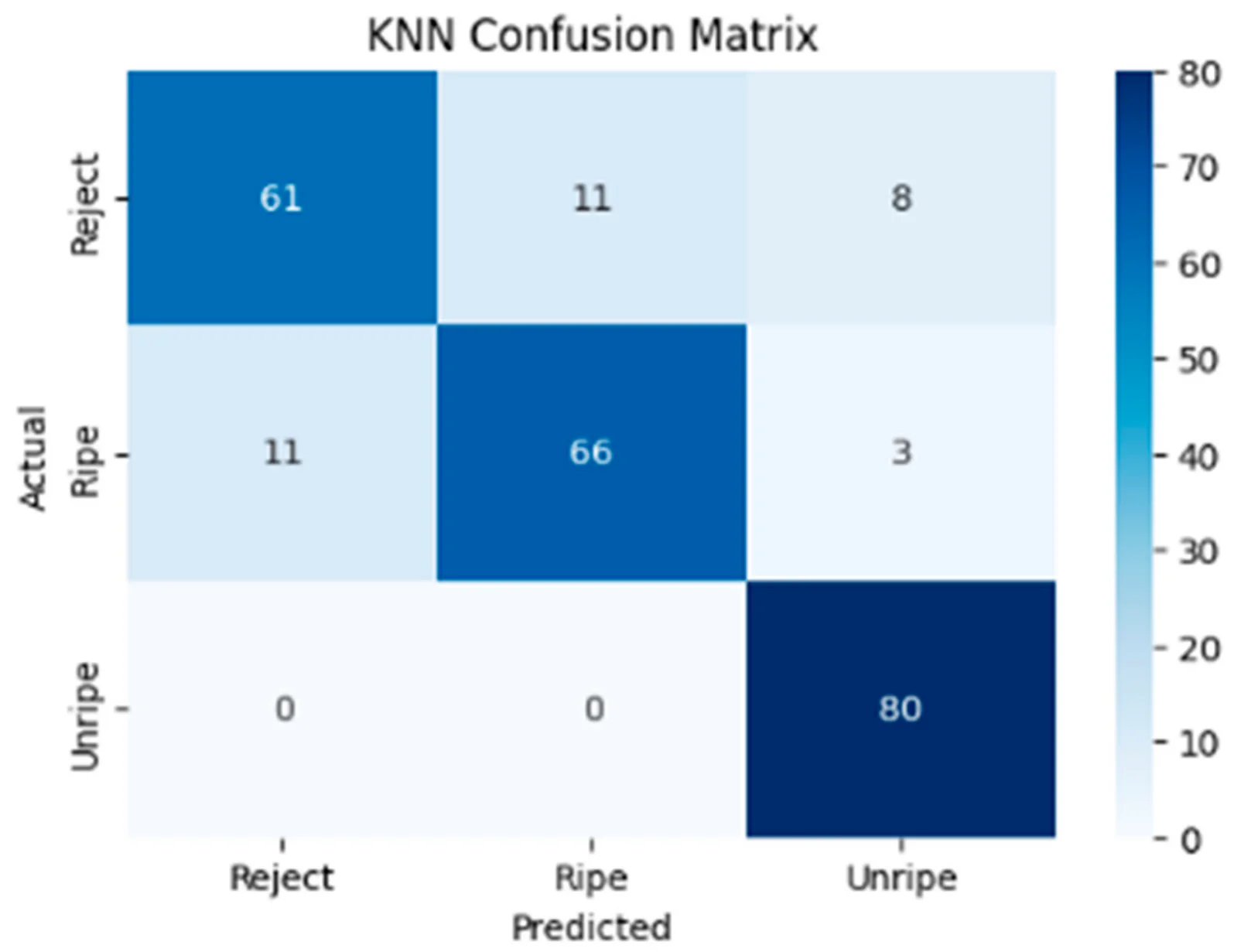
KNN confusion matrix for tomato dataset.

**Figure 10:**
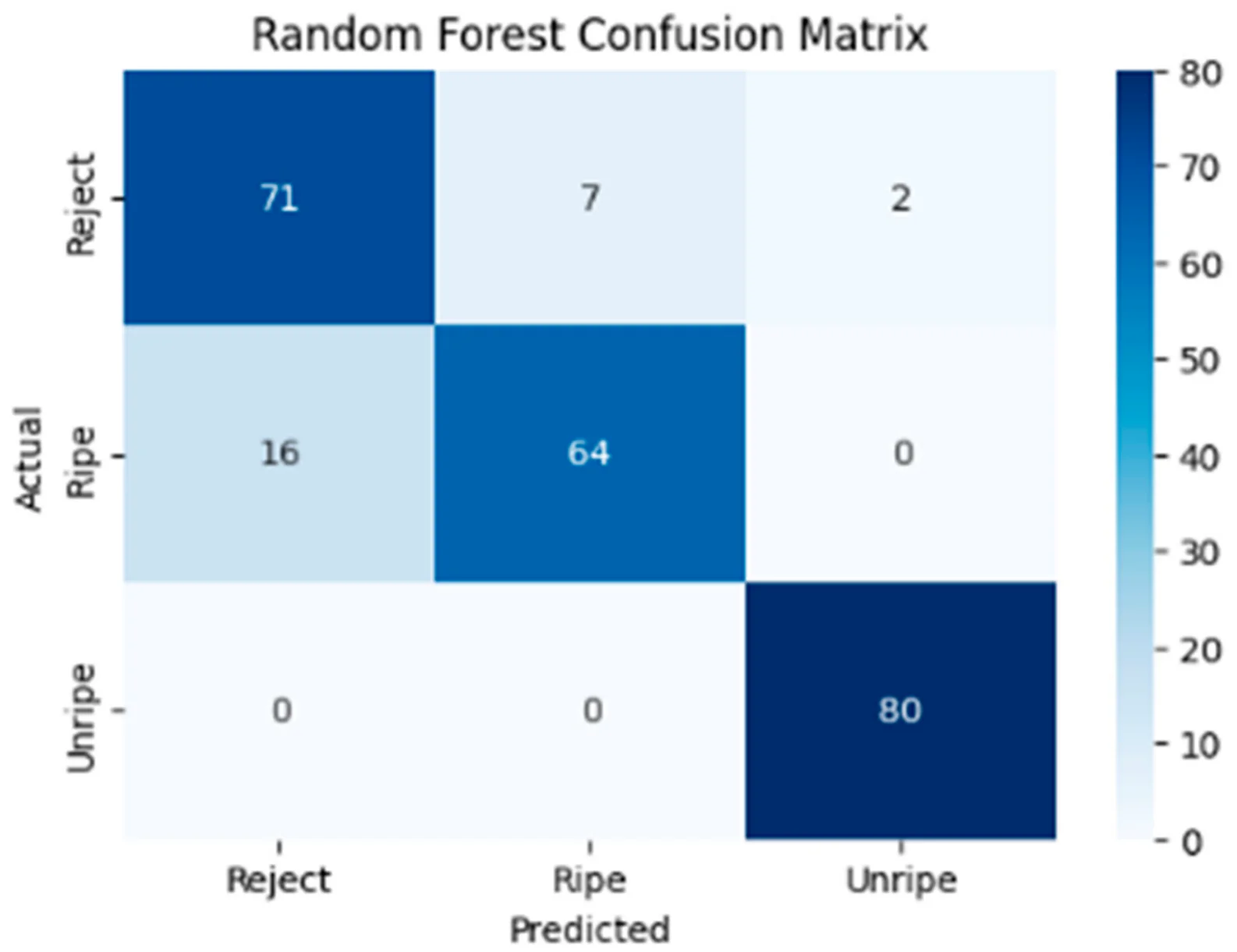
RF confusion matrix for tomato dataset.

**Figure 11:**
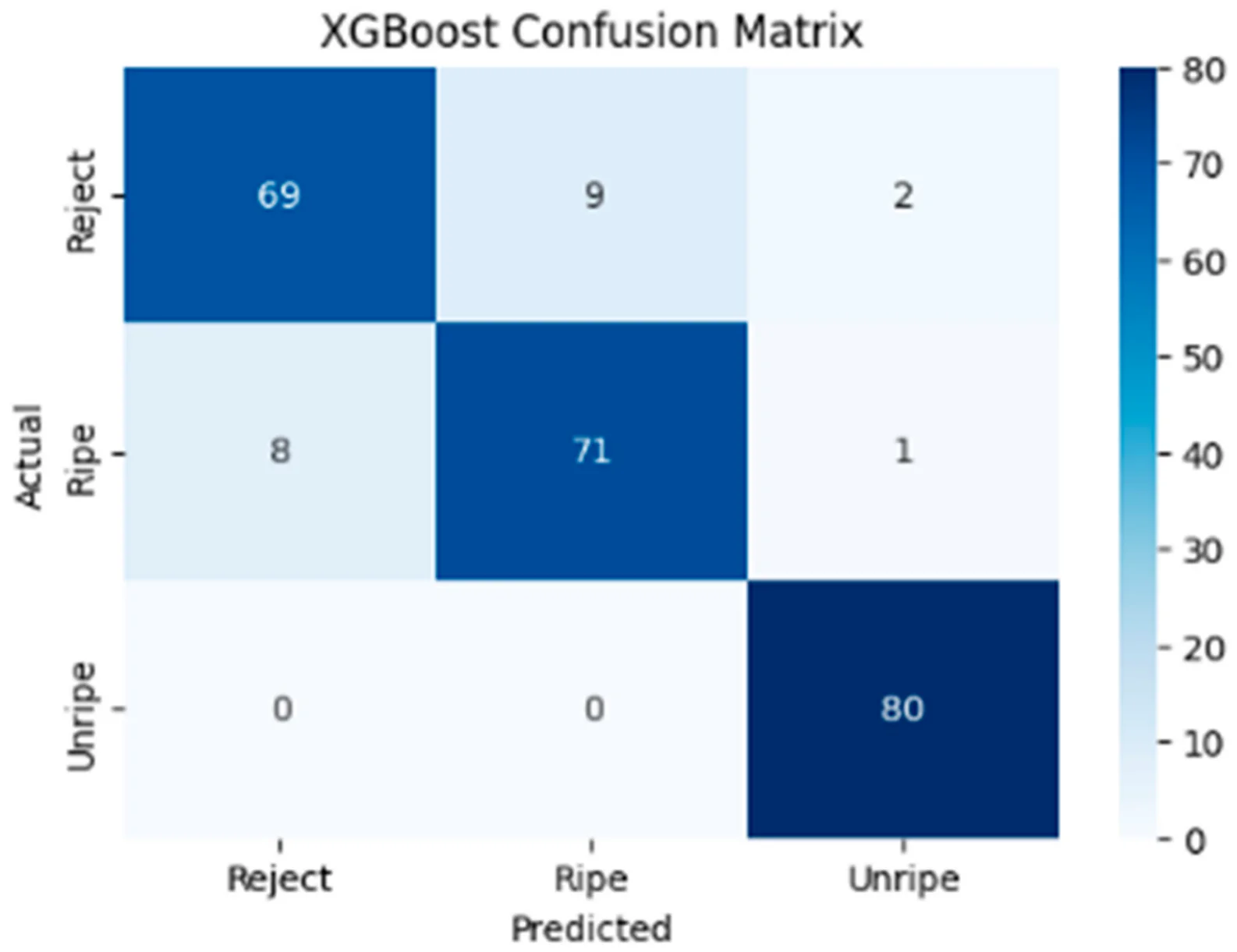
XGBoost confusion matrix for tomato dataset.

Compared to FruitNet, the tomato dataset achieved higher overall accuracies owing to clearer inter-class visual differences. The confusion matrices shown in Figure 9, Figure 10 and Figure 11 reveal that misclassifications were primarily between *Ripe* and *Unripe*, whereas the *Reject* class was identified with strong precision. Among the classifiers, XGBoost performed best, followed closely by RF, whereas KNN struggled with class overlaps.

**Figure 12:**
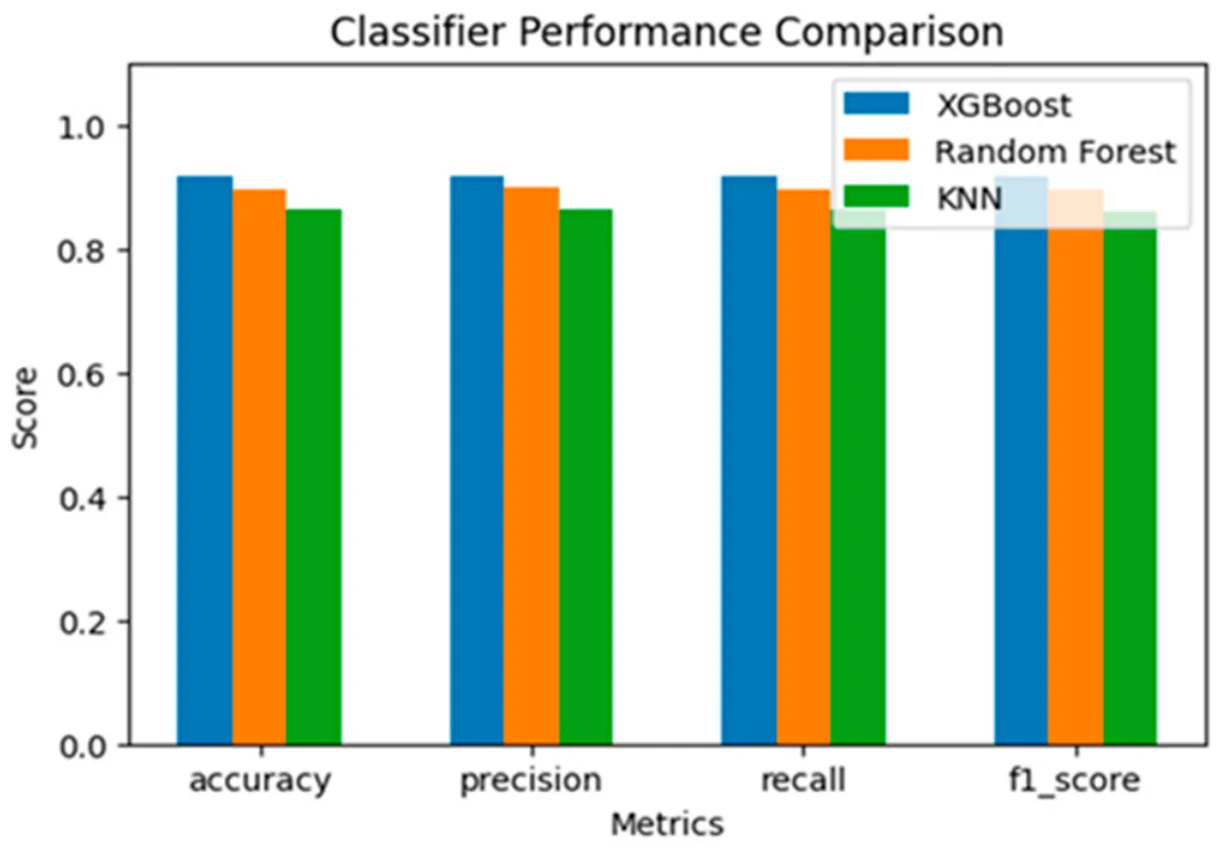
Classifiers’ performance comparison for the tomato dataset.

Figure 12 shows a comparison of all classifiers used in this research to evaluate the tomato dataset.

## 4. Discussion

The results demonstrate that the proposed hybrid framework effectively captured markers related to fruit quality in terms of both high-level semantic information and low-level visual characteristics. The incorporation of deep features extracted through ResNet50V2 with precisely designed descriptors substantially improved the discriminative capability of the feature space. ResNet50V2 was used to capture complex hierarchical patterns linked with fruit appearance, while HOG and LBP descriptors provided detailed information about changes in texture and surface irregularities. The color histogram features enabled the identification of ripeness and discoloration patterns; similarly, shape descriptors helped distinguish fruits affected by physical damage or deformation. Both these feature sets complemented each other effectively and enabled the model to represent fruit quality more comprehensively than a single feature extraction approach. As a result, the combination of both the features produced more precise and reliable classification outcomes across both datasets.

The categorization of different fruit types with varying qualities is a challenging task due to the presence of visually similar categories, as shown by the results obtained on the FruitNet dataset. Especially, it was hard to identify the “Mixed Quality” class because it showed a greater degree of overlap with the “Good” and “Bad” categories, which made it fairly difficult to classify. It was also further endorsed by the confusion matrix analysis which revealed that most classification errors occurred within these neighboring quality classes, rather than between distinctly different categories. This behavior suggests that some fruit samples shared common visual characteristics, resulting in ambiguous decision boundaries. Despite these challenges, the proposed framework was successful in maintaining high classification performance, which indicated that the selected hybrid features were successful in capturing subtle quality-related distinctions. The strong results further confirm the effectiveness of combining deep and handcrafted descriptors for handling complex multiclass classification problems.

Similar trends were observed on the tomato dataset where classification was based on ripeness levels. The tomato dataset showed relatively clearer visual distinctions between classes, as compared to FruitNet, contributing to a better ability to separate. The ripeness-related changes in characteristics like color, texture, and surface appearance were efficiently captured through the extracted feature set. This enabled the classifiers to learn meaningful decision boundaries. The proposed methodology generalized well across different fruit categories, as suggested by the confusion matrix which demonstrated fewer misclassifications among tomato classes. These results highlight the adaptability and flexibility of the framework and how capable it is to handle datasets with distinctive characteristics. The results also indicate that the selected features remained robust despite differences in fruit type, appearance, and quality indicators.

XGBoost was consistent in achieving the highest performance across all evaluation metrics among all the classifiers under consideration. This high performance of XGBoost can be attributed to its boosting mechanism, which allows it to sequentially learn from previous errors and focus on difficult samples during training. Along with the aforementioned features, built-in regularization and effective handling of high-dimensional data enabled XGBoost to make use of the fused feature representation more efficiently than the other classifiers. The results of Random Forest were also competitive and demonstrated robustness due to its ensemble structure; however, it occasionally showed lower precision in classes with limited sample size. The performance of KNN was satisfactory; however, when dealing with complex and high-dimensional hybrid feature spaces, its distance-based classification strategy was less effective. These observations show that for extracting the full predictive potential of the proposed feature representation, advanced ensemble learning approaches are better suited.

Comparison of the current study with existing ones further validates the effectiveness of the proposed approach as shown in Table 5. While several previous research articles were based exclusively on deep learning models or handcrafted feature extraction methods, the proposed framework combines the strengths of both approaches within a unified design. This combined architecture contributed to improved classification accuracy along with enhanced generalization across datasets with different quality assessment criteria. The successful performance achieved using these approaches demonstrates that incorporating complementary feature representations provides a richer understanding of fruit quality characteristics as opposed to relying on a single source of information. Overall, the results confirm that hybrid feature extraction strategies remain a practical and reliable solution for intelligent agricultural applications, especially in scenarios where robustness, interpretability, and classification accuracy are equally important.

## 5. Conclusions and Future Work

This paper proposed a hybrid approach for fruit quality classification by fusing deep learning features from ResNet50V2 with handcrafted descriptors, such as color histograms, shape features, HOG, and LBP. Via feature scaling, ANOVA F-score based selection, and classification using KNN, Random Forest, and XGBoost, the approach showed strong performance across both the FruitNet and tomato datasets. XGBoost attained the highest accuracy and F1-scores, remarkably excelling in classifying visually uncertain classes such as “Mixed Quality” and “Reject” among the models. These results emphasize the efficacy of fusing multi-scale features with robust ensemble methods for practical fruit-grading tasks.

The reliability of the proposed approach was assessed using stratified training, validation, and independent test subsets, with the test data kept separate from model development and used only for final performance evaluation. The consistent classification performance obtained on the independent test subsets provides evidence of the effectiveness of the proposed hybrid feature-based approach on the evaluated datasets. However, the present study was evaluated using the available FruitNet and tomato datasets, and no additional external dataset was used for independent cross-dataset validation.

Future work may focus on evaluation of this approach on independent external datasets collected under different environmental and acquisition conditions to further assess the robustness and generalizability of the proposed methodology. In future, this pipeline can also be used in real time on edge devices for real use in farms and sorting facilities. Another hopeful direction is the incorporation of this system into agricultural supervising frameworks to support automated quality checks during harvesting and packaging processes. Overall, this paper lays the foundation for scalable and intelligent fruit assessment solutions powered by accessible, AI-driven methods.

## Figures and Tables

**Table 1:** Comparison of existing studies.

Ref	Year	Authors	Technique	Dataset(s)	Accuracy (%)
[20]	2019	Bhargava and Bansal	ANN, SVM, SRC	Apple, Banana, Avocado, Orange	95.72
[21]	2020	Bhargava and Bansal	SVM	Two Apple Datasets	96.81, 93.00
[22]	2020	Long and Thinh	RF, LDA, SVM, KNN	Mango	98.1
[23]	2019	Nasiri et al.	VGG16	Date Fruit (Shahani)	96.98
[24]	2020	Fan et al.	ML + Preprocessing (RF, NB)	Apple	96.5
[26]	2021	Foong et al.	ResNet50	Banana, Apple, Orange	98.89
[27]	2021	Miah et al.	Inception V3	Apple, Mango, Banana, Plum and Orange (Fresh/Rotten)	97.34
[28]	2021	Al Haque et al.	CNN	Banana	98.3
[29]	2025	Sharma and Kumar	ResNet50	Mixed Fruits (Fresh/Rotten)	95.00
[30]	2023	Zha et al.	ResNet50 + SVM	Red Grapes (3-Class Grading)	95.08
[31]	2023	Knott et al.	Dino ViT + Shallow Classifiers (KNN, LR, SVM, RF, XGBoost, MLP)	Apple, Banana	90.00
[32]	2024	Mputu et al.	CNN + SVM (Hybrid)	Tomato Quality	96.67
[33]	2024	Mallegowda et al.	VGG16	Mixed Fruits	90.00
[34]	2024	Tai et al.	ML Algorithms, Ensemble Learning Method	Mango	98.63
[35]	2025	Akram et al.	CNN + Sorting Hardware	Mango, Tomato	95.00, 94.00

**Table 2:** Augmentation techniques.

Technique	Range/Type	Purpose
Rotation	±20°	Accounts for angled fruit orientation
Width Shift	±10%	Off-centered position handling
Height Shift	±10%	Off-centered position handling
Shear	±10%	Compensation for perspective deforming
Zoom	±10%	Studies multi-scale features
Horizontal Flip	Enabled	Ensures balanced robustness
Fill Mode	Nearest-neighbor filling	Maintains edge features after shift

**Table 3:** Classification results on FruitNet dataset.

Classifier	Accuracy	Precision	Recall	F1-Score
XGBoost	97.3%	97.3%	97.3%	97.3%
K-Nearest Neighbors	96.2%	96.2%	96.2%	96.1%
Random Forest	95.4%	95.4%	95.4%	95.4%

**Table 4:** Classification results on the tomato dataset.

Classifier	Accuracy	Precision	Recall	F1-Score
XGBoost	92%	91.6%	91.6%	91.6%
Random Forest	89.5%	89.7%	89.5%	89.5%
K-Nearest Neighbors	86.2%	86.1%	86.2%	89.5%

**Table 5:** Comparison of proposed work with existing studies.

Ref	Year	Technique	Dataset(s)	Accuracy
[23]	2019	VGG16	Date	96.98%
[30]	2023	ResNet50 + SVM	Red Grapes	95.08%
[32]	2024	CNN + SVM	Tomato	96.67%
[35]	2025	CNN + Sorting Hardware	Mango, Tomato	95.00%, 94.00%
Proposed Model	2025	ResNet50V2 + HOG + LBP + Color and Shape Features + XGBoost	FruitNet Dataset (Apple, Banana, Guava, Lime, Orange, and Pomegranate), Tomato Dataset	97.3%, 92.00%

## Data Availability

The original contributions presented in the study are included in the article, further inquiries can be directed to the corresponding author.
